# The monoclonal antibody VYD2311 potently neutralizes dominant and emerging SARS-CoV-2 variants

**DOI:** 10.1172/jci.insight.208557

**Published:** 2026-08-11

**Authors:** Ian A. Mellis, Madeline Wu, Kristin Daniel, Hsiang Hong, Yicheng Guo, David D. Ho

**Affiliations:** 1 Aaron Diamond AIDS Research Center and; 2 Department of Pathology and Cell Biology, Columbia University Vagelos College of Physicians and Surgeons, New York, New York, USA.; 3 Department of Microbiology, National Taiwan University College of Medicine, Taipei, Taiwan.; 4 Division of Infectious Diseases, Department of Medicine, and; 5 Pandemic Research Alliance unit at the Wu Center for Pandemic Research, Columbia University Vagelos College of Physicians and Surgeons, New York, New York, USA.

**Keywords:** Infectious disease, Public Health, COVID-19

## Abstract

Mellis and colleagues report that a laboratory-synthesized version of VYD2311, an antibody in trials for preventing COVID-19, inhibits many SARS-CoV-2 variants in laboratory-based models of infection

**To the Editor:** For millions of immunocompromised individuals, vaccines may not elicit adequate protection from infections, so alternative strategies for pre-exposure prophylaxis are essential. There is only 1 nonvaccine product authorized in the U.S. as pre-exposure prophylaxis against COVID-19: pemivibart (Invivyd). Pemivibart is a monoclonal antibody targeting the SARS-CoV-2 spike protein receptor–binding domain class 1/4 region, which was authorized in March 2024, when the JN.1 variant was dominant (1). However, SARS-CoV-2 has continued to evolve, and we previously showed that pemivibart had lower neutralizing activity in vitro against recent SARS-CoV-2 variants, such as KP.3.1.1, NB.1.8.1, and LP.8.1.1, than against JN.1 (2, 3). In 2024, Invivyd began clinical testing of a new monoclonal antibody, VYD2311, and subsequently initiated a phase 3 clinical trial to test it as pre-exposure prophylaxis against COVID-19 (4). VYD2311 was derived from pemivibart, containing 6 mutations (3 in the heavy chain and 3 in the light chain; Supplemental Figure 1; supplemental material available online with this article; https://doi.org/10.1172/jci.insight.208557DS1). In a patent, Invivyd reported that VYD2311 had high neutralizing potency against recent JN.1 subvariants, including KP.3.1.1 and LP.8.1, but they did not report results for currently dominant and expanding variants. As of April 2026, SARS-CoV-2 variants XFG and NB.1.8.1 are dominant around the world, and the genetically distant variant BA.3.2.2 is emerging in several countries (Supplemental Figure 2) (5–7). It is essential to monitor the efficacy of antibody products in the setting of evolving viral variants.

Using Vesicular Stomatitis Virus–based (VSV-based) pseudovirus neutralization assays in Vero-E6 cells, we measured the neutralizing activities of versions of VYD2311 and pemivibart synthesized in our laboratory against recent, dominant, and expanding SARS-CoV-2 variants: D614G (representing the ancestral strain), JN.1, KP.3.1.1, LP.8.1.1, NB.1.8.1, XFG, and BA.3.2.2 (Figure 1) (3). The 50% inhibitory concentrations (IC_50_) of laboratory-synthesized VYD2311 against JN.1, XFG, BA.3.2.2, and NB.1.8.1 were approximately 0.004, 0.002, 0.013, and 0.126 μg/mL, respectively. The 90% inhibitory concentrations (IC_90_) of VYD2311 against JN.1, XFG, BA.3.2.2, and NB.1.8.1 were approximately 0.029, 0.030, 0.126, and 1.05 μg/mL, respectively. Comparing laboratory-synthesized VYD2311 to laboratory-synthesized pemivibart, the IC_50_ against JN.1, XFG, BA.3.2.2, and NB.1.8.1 were approximately 27, 51.5, 31.54, and 123.6-fold lower, respectively, with even larger factor changes in IC_90_ (Supplemental Figure 3).

Pemivibart is authorized to be administered i.v. every 3 months at a dose of 4,500 mg and was reported to have a trough serum concentration of approximately 175 μg/mL. Adintrevimab, the parental molecule to pemivibart, was tested in Phase 1 trials at 300 mg or 600 mg i.m. doses or at a 500 mg i.v. dose. The mean serum adintrevimab concentration 90 days after administration in a peer-reviewed study was > 10 μg/mL for all 3 administration methods (8). The VYD2311 patent also reports Phase 1/2 testing of 1,000 mg i.m., 1,250 mg s.c., and 2,000 mg or 4,500 mg i.v. VYD2311 doses, which led to mean serum concentrations of > 10 μg/mL at 90 days after administration (or 45 days for s.c. dosing) (5). Therefore, if VYD2311 pharmacokinetics are confirmed and similar to adintrevimab, then at the reported doses, VYD2311 could circulate in the blood at higher than the IC_99_ values of all tested variants for at least 90 days (Supplemental Figure 3).

These data show that pemivibart has lower neutralizing activity against evolving SARS-CoV-2 variants of concern, including NB.1.8.1 and BA.3.2.2, and this raises concerns about the continued efficacy of pemivibart as pre-exposure prophylaxis against COVID-19. Future mechanistic studies will be required to explain the causes of lower neutralizing activity against these variants. However, reassuringly, these results also show that VYD2311 potently neutralizes all tested SARS-CoV-2 variants, including those that are most prevalent worldwide, XFG and NB.1.8.1, and the genetically and antigenically distant emerging variant BA.3.2.2. Due to the breadth and potency of its neutralizing activity and its reported pharmacokinetics, VYD2311 is a promising investigational product for the prevention and treatment of COVID-19 in immunocompromised individuals. These results again show that it is critical to continuously monitor the neutralizing activity and clinical efficacy of monoclonal antibody agents directed against evolving pathogens.

## Conflict of Interest

DDH co-founded TaiMed Biologics and RenBio, and he serves as a consultant for WuXi Biologics and Brii Biosciences and is a board director at Vicarious Surgical. The remaining authors declare no conflicts of interest. None of the authors hold equity in, receive payments from, or consult for Invivyd.

## Funding support

This work is the result of NIH funding, in whole or in part, and is subject to the NIH Public Access Policy. Through acceptance of this federal funding, the NIH has been given a right to make the work publicly available in PubMed Central.

- NIH SARS-CoV-2 Assessment of Viral Evolution (SAVE) Program (subcontract no. 0258-A700-4609 under federal contract no. 75N93021C00014) to David D. Ho
- The Bill and Melinda Gates Foundation (project no. INV019355) to David D. Ho
- Internal startup funding (UR014016) from Columbia University to Yicheng Guo

## Supplementary Material

Supplemental data

Supporting data values

## Figures and Tables

**Figure 1:**
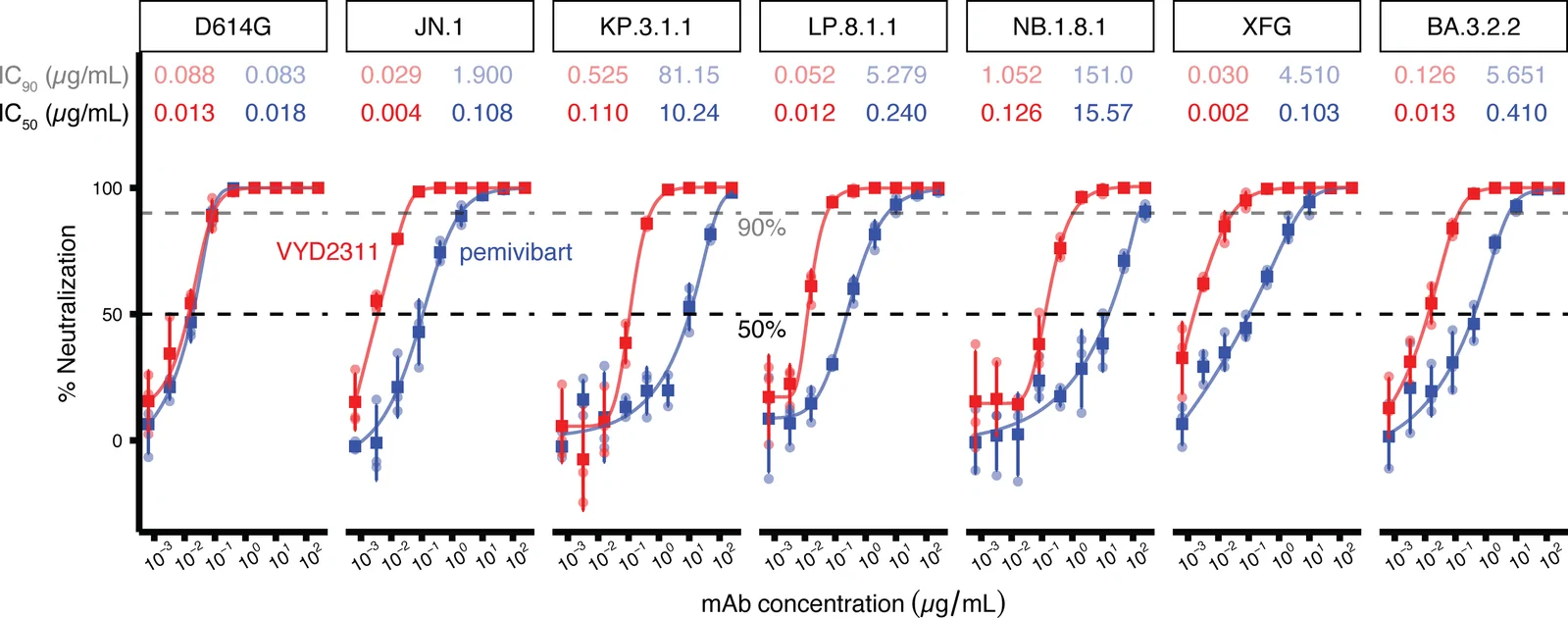
Neutralizing activity of research-grade VYD2311 and research-grade pemivibart. Percent neutralization of the indicated pseudotyped viruses by VYD2311 (red) and pemivibart (blue). Dots indicate technical replicates, squares indicate mean, I bars indicate standard deviation. Five-fold serial dilutions from a maximum tested concentration of 250 μg/mL, 9 doses tested. Dashed lines indicate 50% and 90% neutralizations. The indicated inhibitory concentrations (ICs) of each antibody at 50% and 90% neutralization levels against each variant are annotated above each pair of curves.

## Version 1 08/11/2026

In-Press Preview

## Version 2 09/22/2026

Electronic publication
